# Editorial: Enhancing woody plant growth and resilience through nature-based solutions

**DOI:** 10.3389/fpls.2026.1852603

**Published:** 2026-05-12

**Authors:** Carmen Arena, Maryam Teimouri, Mehrdad Zarafshar

**Affiliations:** 1 Department of Biology, University of Naples Federico II, Naples, Italy; 2 Agricultural Research Education and Extension Organization (AREEO), Research Institute of Forests and Rangelands, Tehran, Iran; 3 Department of Forestry and Wood Technology, Faculty of Technology, Linnaeus University, Växjö, Sweden

**Keywords:** arbuscular mycorrhizal fungi, climate change, forest soil, leaf functional traits, soil nutrients, woody species

Nature-based solutions (NbS) are increasingly recognized as effective strategies to enhance plant growth and resilience for counteracting the ongoing environmental challenges. In a context dominated by climate change, land degradation, and biodiversity loss, woody plants play a crucial role in ecosystem functioning, carbon sequestration, and landscape stability. Hence, improving their performance through sustainable and eco-friendly approaches is a key priority for both research and practical applications.

The Research Topic “Enhancing Woody Plant Growth and Resilience Through Nature-Based Solutions” was conceived to explore innovative and multidisciplinary approaches aimed at strengthening plant performance while minimizing environmental impact. The 15 contributions collected in this Topic reflect the diversity of current research efforts, spanning physiological, biochemical, ecological, and applied perspectives. Together, these studies provide valuable insights into how NbS can be integrated into plant science to support resilience and sustainability.

The contributions collected in this Research Topic reflect the multifaceted nature of plant resilience, highlighting the importance of integrating physiological, ecological, and management-based approaches.

A first group of studies focuses on plant responses to environmental stress, particularly drought and climate variability. Che et al. demonstrated that drought, especially during the growing season from April to August, represents the main limiting factor for the radial growth of *Caragana korshinskii*, and that targeted management strategies such as supplementary irrigation and slope positioning can mitigate these limitations. Similarly, Wang et al. showed that species mixing ratios (e.g., Pinus–Quercus combinations) strongly influence drought resistance and resilience in temperate forests, emphasizing that optimal combinations of species can enhance ecosystem stability under prolonged water deficit. At a broader scale, Zheng et al. highlighted the importance of climate legacy effects, demonstrating that previous-year moisture conditions significantly influence vegetation productivity than current-year conditions across large-scale ecosystems.

Closely related to climate responses, several studies explored plant performance and ecosystem functioning under changing environmental conditions. Sun et al. assessed the functional vulnerability of tropical woody plant communities, revealing high spatial heterogeneity and a dominant role of soil properties and trait distributions across spatial scales. In parallel, Wang et al. investigated net ecosystem carbon change (NECC), showing that forest productivity is co-regulated by stand characteristics, structural diversity, and environmental factors, with their relative influence shifting across developmental stages. These findings underline the importance of considering ecosystem-level processes when evaluating plant resilience.

Another important set of contributions addresses plant–soil interactions and nutrient dynamics as key drivers of growth and resilience. Wang et al. demonstrated that phosphorus addition and arbuscular mycorrhizal fungi (AMF) inoculation modify rhizosphere nutrient availability (e.g., available phosphorus, soil organic carbon) and microbial community composition and diversity in karst plantation systems.

Similarly, Ouyang et al. highlighted how stand structure and developmental stage regulate nitrogen and phosphorus resorption efficiency across organs, revealing coordinated aboveground–belowground nutrient-use strategies that vary across developmental stages. In addition, Wang et al. showed that phosphorus fertilization (up to 500 g·plant^-^¹) enhances early growth, biomass accumulation, and nitrogen–phosphorus content in *Pinus massoniana* hybrid saplings, emphasizing the interaction between genotype and nutrient availability. Plant adaptation strategies were further explored through morphological and functional traits. Wang et al. analyzed fine root morphological traits and biomass distribution across species growing in coastal saline soils, demonstrating species-specific strategies linked to soil salinity, moisture, and nutrient availability. At the genetic and biotechnological level, Bharati and Severová highlighted the potential of artificial polyploidy as a tool to induce chromosome duplication, enhancing growth performance and resistance to both biotic and abiotic stresses in woody species.

To further enrich the landscape of NbS, biodiversity-related processes were also investigated within this Research Topic. Wang et al. showed that Moso bamboo invasion alters forest structure by reducing tree-layer diversity while modifying soil nutrient cycling and increasing microbial carbon limitation, thus reshaping ecosystem functioning. Complementarily, Wang et al. proposed an innovative framework integrating dark diversity, functional traits, and diagnostic species to identify stage-specific bottlenecks in forest recovery and guide targeted restoration interventions.

Finally, several studies highlight the application of nature-based solutions in practical contexts. Jiang et al. demonstrated how different support structures (e.g., bamboo poles vs. climbing nets) influence biomass allocation and growth performance in vertical greening systems, offering practical insights for urban sustainability. Zhang et al. combined species distribution models and growth traits to predict future timber production zones under climate change scenarios, identifying shifts in suitable habitats and productivity. Finally, Mueller et al. emphasized the importance of adaptive forest management strategies along infrastructure corridors, identifying woody species capable of maintaining protective ecosystem services under future climate conditions.Overall, these contributions collectively demonstrate that enhancing woody plant growth and resilience requires an integrative approach that combines physiological understanding, ecological interactions, and sustainable management practices across scales.

In conclusion, the contributions assembled in this Research Topic highlight the growing importance of integrating nature-based solutions into plant science. Despite their diversity, all studies converge on a common perspective: enhancing plant resilience requires a all-inclusive understanding of physiological processes, ecological interactions, and sustainable practices.

Future research should aim to further integrate these approaches, combining experimental, ecological, and technological perspectives. Such integration will be essential to address the challenges posed by climate change and to ensure the sustainable management of woody plant systems.

As Topic Editors, we believe that the insights provided in this Research Topic will stimulate further research and contribute to the development of innovative and sustainable solutions for plant growth and resilience.

